# Commentary: The dynamics of foreign language enjoyment: An ecological momentary assessment

**DOI:** 10.3389/fpsyg.2022.993769

**Published:** 2022-09-16

**Authors:** Ting Hu, Huan Mei

**Affiliations:** ^1^Department of Humanities and Social Sciences, Zhejiang Conservatory of Music, Hangzhou, China; ^2^School of English Studies, Shanghai International Studies University, Shanghai, China

**Keywords:** foreign language enjoyment, dynamics, ecological momentary assessment, dimensions of FLE, drivers of FLE

## Introduction

The recent paper by Elahi Shirvan et al. (2020) has drawn significant scholarly attention to foreign language enjoyment (FLE), a critical yet understudied topic in second language acquisition research (Dewaele and MacIntyre, 2016; Boudreau et al., 2018). Employing ecological momentary assessment (EMA) to measure FLE across four hierarchical timescales, the authors validated two dimensions of FLE identified in previous studies: *FLE-Private* and *FLE-Social* (Dewaele and MacIntyre, 2014, 2016), confirmed the factors of “self,” “self-peer,” “self-teachers” and “self-peer-teacher” as drivers of FLE (Dewaele and MacIntyre, 2019), and revealed contextualized intra- and inter-variations of it from a complexity dynamic systems perspective. Given its theoretical, methodological and practical contributions, we believe it a worthwhile endeavor to comment on and introduce it to a wider audience.

## Theoretical contribution

The study's greatest theoretical contribution is its findings on the dimensions, drivers and dynamics of FLE, which facilitate a richer understanding on the nature of FLE, its conceptualization and theorization. We argue that, based on the private and social perspectives adopted by this study toward the construct, a worthy research agenda is to further explore the relationship between them. Answers to such questions as “Are these two dimensions correlated or independent?” and “Does an increase in private FLE concur with a drop or increase in the social FLE?” would certainly lend more clarity as to the nature of FLE and ultimately, as rightly suggested by the authors, help researchers “come up with a comprehensive model of FLE” (Elahi Shirvan et al., 2020, p. 13).

## Methodological innovation

Credit should also be given to the authors for the methodology. To start with, the paper showcases the potential of EMA for research on second language learning and teaching. EMA, also known as the experience sampling method (Scollon et al., 2003), refers to “a research procedure for studying what people do, feel, and think during their daily lives” (Larson and Csikszentmihalyi, 2014, p. 21). This methodology collects *in situ* self-reports repeatedly and systematically in the contexts of day-to-day activities (Stone et al., 1999). Originally employed in psychology, it is only recently that it has attracted attention in second language acquisition (SLA) research (Hiver and Al-Hoorie, 2019; Arndt et al., 2021). Elahi Shirvan et al. (2020) succeeded in demonstrating the strengths of EMA and how this new method is well-suited to study second language emotions, such as foreign language enjoyment (FLE). This paper has convincingly exemplified that EMA, a method of higher validity and with more accurate information due to its immediacy (Shiffman et al., 2008; Ellison et al., 2020), can be an effective complement to retrospective methods such as interview, which usually suffer from recall biases (Arndt et al., 2021). Moreover, as argued by the authors, foreign language learners are “persons-in-context” (Ushioda, 2009), and in light of the complex dynamic systems theory, learner emotions were found to be dynamic, contingent on and interactive with situational affordances (Dewaele and Dewaele, 2017; Elahi Shirvan and Talebzadeh, 2017; Boudreau et al., 2018). As a method that can shed light on the dynamics of behavior in authentic contexts (Shiffman et al., 2008), EMA thus uniquely allows researchers to track the dynamics of real-life emotions as a consequence of “changing proximal stimuli (Zohar et al., 2003; Fisher and Noble, 2004), personality traits (Grandey et al., 2002), and perceptions of job properties (Fisher, 2002)” (Elahi Shirvan et al., 2020, p. 4). In other words, it enables the study of emotions from an ecological perspective and is therefore appropriate for the investigation of FLE.

Secondly, in line with Wang et al.'s (2021) call for expanding the range of time frames in SLA research, this paper used EMA to chart enjoyment across four hierarchical timescales, thus presenting readers with different pieces of the enjoyment jigsaw. In the investigation of this complex system of FLE, a salient attribute of which is the momentary and situational changes of learners' systems, “moment” was seen as a critical variant. Consequently, as pointed out by the authors, the dynamicity of FLE can not be captured through single-shot measures alone, such as questionnaires, popular in foreign language emotion research (Li et al., 2021), but rather, can only be observed through “data that is dense (i.e., collected at many regular measurement points), and longitudinal (i.e., collected over a longer period of time)” (van Dijk et al., 2011, p. 62). Thus, this study employed a time-based sampling scheme of EMA, in which the authors operationalized the concept of time into seconds, minutes, weeks and months, and measured participants' enjoyment of these four temporal resolutions with the idiodynamic method, enjoymeter, journal and interviews respectively.

In addition, since EMA data were reported in-class and while students were using English, the data provided important information on the interactions between enjoyment and the context, namely, the classroom setting, learners' self, teacher-student relationships and peer relationships. It was thus found both internal and external ecological factors, that is, factors associated with “self,” “self-peer,” “self-teachers” and “self-peer-teacher” significantly influence FLE.

In sum, by employing EMA, this paper not only echoes Dewaele and Li's (2020) call for original methodological approaches for research on FLE, but also highlights the method's unique value in tracing the dynamic patterns and individual-environment interactions of foreign language emotions in general, as well as in providing a more accurate picture of participants' actual feelings. By utilizing this innovative method, this study advances the positive psychology movement in the field of SLA in more ways than one. Firstly, it demonstrated the feasibility and advantage of the cross-disciplinary endeavor of applying appropriate methods from neighboring disciplines to SLA research. Additionally, the use of this method can be further used to generate a more comprehensive picture of learner differences, as it can be applied to explore other positive psychology factors in SLA besides FLE (Dewaele and Li, 2020), such as resilience, wellbeing, resilience, and engagement (Wang et al., 2021).

## Practical implications

Another key highlight of this paper is the practical implications that teachers and teacher trainers can draw on. Findings from this paper can help teachers design more nuanced teaching models and activities that boost FLE, and be of value to teacher training. Corroborating previous findings on the positive relationship between favorable attitudes toward the teacher and FLE (Dewaele et al., 2017), this study underscores the crucial influence that teachers have on their students' FLE. Given that “self-peer,” “self-teachers” and “self-peer-teacher” factors affect FLE, and since what teachers do in classrooms is strongly linked to FLE (Dewaele et al., 2017), it is necessary for teachers to, for instance, increase students' interest and engagement (Wang et al., 2021), incorporate collaborative activities to foster learners' interactions, show respect and encouragement to students, and create a friendly classroom environment that nurtures positive student-student and student-teacher relationships. As reported in the cases of Sara and Sanaz in this study, some students' enjoyment may be more prone to influence from *FLE-private* factors, while others, *FLE-social* ones. Thus, teachers need to be more attentive to individual differences in FLE, and customize their teaching methods accordingly. Building on this study and previous literature, researchers can carry out intervention studies to explore which pedagogical techniques are most effective in enhancing FLE in any given scenario. Findings from such studies are rewarding and informative for both teaching practices and pre- and in-service teacher training.

## Discussion

All these merits notwithstanding, we have two reservations about this study. To start with, it could have benefited from a combination of emic and etic perspectives than only the former. With idiodynamic method, EMA, data from journal and interview, this paper adopted a predominantly emic perspective to study FLE, in other words, the perspective from the inside (Morris et al., 1999). All of the aforementioned data collection methods rely heavily on participants' voices through self-reports. However, used independently, it has inherent shortcomings, such as social desirability bias, that is, participants tend to give responses that put them in a socially favorable light instead of ones that reflect their true feelings (Nederhof, 1985). Hence, complementing it with an etic perspective, namely, the perspective from outside, such as classroom observation on students' FLE can help mitigate these weaknesses and provide a more balanced view of FLE. Secondly, the study could have been more persuasive had it provided a clearer rationale for incorporating data collection tools for analysis of FLE on the four timescales. This would contribute to replication studies and ultimately advancement of research on FLE.

In conclusion, Elahi Shirvan et al.'s (2020) study constitutes a good source of inspiration on the theorization of FLE, methodological innovation and practical implications of research on FLE. We further this line of inquiry by advocating future research on the relationships between the dimensions within FLE, and intervention studies that focus on effective practices to boost FLE. Therefore, this paper is a good start, hopefully leading to more a fruitful and in-depth discussion in the promising research area of FLE.

## Author contributions

TH and HM drafted the manuscript. TH did the revisions. Both authors contributed to the manuscript and approved it for publication.

## Funding

This study was funded by Department of Education of Zhejiang Province (G001A3202208).

## Conflict of interest

The authors declare that the research was conducted in the absence of any commercial or financial relationships that could be construed as a potential conflict of interest.

## Publisher's note

All claims expressed in this article are solely those of the authors and do not necessarily represent those of their affiliated organizations, or those of the publisher, the editors and the reviewers. Any product that may be evaluated in this article, or claim that may be made by its manufacturer, is not guaranteed or endorsed by the publisher.
